# Coupling and Slips in Photosynthetic Reactions—From Femtoseconds to Eons

**DOI:** 10.3390/plants12223878

**Published:** 2023-11-16

**Authors:** Nathan Nelson

**Affiliations:** Department of Biochemistry and Molecular Biology, The George S. Wise Faculty of Life Sciences, Tel Aviv University, Tel Aviv 69978, Israel; nelson@tauex.tau.ac.il

**Keywords:** photosynthesis, coupling, slip, membrane complexes, time, rates, order, disorder

## Abstract

Photosynthesis stands as a unique biological phenomenon that can be comprehensively explored across a wide spectrum, from femtoseconds to eons. Across each timespan, a delicate interplay exists between coupling and inherent deviations that are essential for sustaining the overall efficiency of the system. Both quantum mechanics and thermodynamics act as guiding principles for the diverse processes occurring from femtoseconds to eons. Processes such as excitation energy transfer and the accumulation of oxygen in the atmosphere, along with the proliferation of organic matter on the Earth’s surface, are all governed by the coupling–slip principle. This article will delve into select time points along this expansive scale. It will highlight the interconnections between photosynthesis, the global population, disorder, and the issue of global warming.

## 1. Introduction

Living systems are essentially open systems that maintain continuous energy and matter exchange with the environment. Survival is based on a process known as negative entropy. When a system has enough negentropy (negative entropy), it can maintain an organized state. The timescale of reactions involved in life ranges from femtoseconds to eons. Quantum techniques are employed to explain fast reactions and energy exchange in highly rapid reactions on sub-nanosecond timescales, involving quantum coherence. Photosynthesis serves as a prime example of this phenomenon.

Photosynthesis spans the widest range of redox potential in the biochemistry of life. Operating under extreme redox potential poses challenges, including protection against damage from highly reactive components—such as singlet oxygen, a by-product of photochemical activity—and prevention of the loss of high-energy electrons to nonproductive components in the environment. In all photosynthetic systems, the conversion of light into chemical energy relies on electronic couplings that facilitate efficient energy transport from light-capturing antenna pigments to the reaction center [1,2,3].

This energy transfer does not simply occur as a cascade of stepwise events down the energy ladder. Instead, it depends on the spatial properties of the delocalized excited-state wavefunctions of the entire pigment–protein complex. Quantum mechanical properties play a role in the highly rapid excitation transfer to the reaction center [4,5,6]. Although nature generally avoids wasteful reactions, some leaks and slips are used to control production rates, which are sometimes necessary for overall reaction efficiency. Like other biological coupled reactions, excitation transfer is susceptible to built-in slips that contribute to the overall process’s efficiency [7]. Slips are processes that occur within biological systems to control processes by reducing net productivity.

## 2. Femtosecond to Millisecond Coupling and Slips

The reaction velocities of photosynthetic processes span the broadest time range known in biochemistry. When looking at the velocity of light absorbance, transfer of electrons in photochemical reactions, electron transfer through the Photosystem I (PSI) complex, transfer of electrons from P700 to ferredoxin, electron transfer chain and ATP synthesis, carbon fixation, and the export of stable products, we find reaction times in femtoseconds (10^−15^), picoseconds (10^−12^), nanoseconds (10^−9^), microseconds (10^−6^), milliseconds (10^−3^), and seconds, respectively. The mechanism of each reaction depends on its respective time domain. The photophysical and photochemical reactions that occur between femtoseconds and nanoseconds are governed by quantum mechanics. Both classical and quantum electrodynamics predict the existence of dipole–dipole long-range electrodynamic intermolecular fields, which can be observed and calculated [8]. Measurements using two-dimensional electronic spectroscopy (2DES) have shown that the initial dynamic response of photosynthetic proteins involves quantum coherence [6]. Quantum decoherence, the loss of quantum coherence, occurs as information from a system is lost to the environment. Reactions that occur between microseconds and seconds are governed by electrostatics and statistical mechanics [9]. As early as 40 years ago, Shuvalov and Parson [10] were able to demonstrate slippages in bacterial photosynthetic reaction centers using picosecond spectroscopic studies. Arguably, all imperfections occurring on the femtosecond timescale can be characterized as decoherence and understood as mechanistic deviations. Decoherence can be understood as the information loss from a system to its environment, as dictated by environmental disorder (heat), in line with the principles of the second law of thermodynamics [11]. Consequently, not every photon absorbed by a photosynthetic system leads to the productive excitation of electrons; some of these excitations might have detrimental effects. Alternative explanations, such as the fluctuation–dissipation theorem in nonequilibrium steady states, were suggested [12]. Explanations and reservations about using decoherence were discussed by C. Negulescu in “Decoherence rhapsody in the photosynthesis process” [13]. I am fully aware that the role of quantum coherence in room-temperature photosynthetic reactions is controversial. I took the liberty of framing it in terms of its practical definition, where electrons or photons exist in a state that allows them to exhibit wave-like behavior and maintain phase relationships. Accordingly, decoherence is a phenomenon that interferes with phase relationships.

Two-dimensional electronic spectroscopy has proved itself to be one of the most powerful tools for studying the excitation dynamics in photosynthetic complexes [14,15]. Quantum coherence was demonstrated in isolated cyanobacterial Photosystem I (PSI) containing 88 chlorophyll a molecule using this technique [6,16]. Based on these observations and several others, we can infer that quantum coherence plays a role in the light absorption and excitation transfer processes of all photosynthetic reaction centers.

The core of every photochemical reaction center is a dimeric structure, and its evolution probably began with a homodimeric structure and progressed from symmetry through pseudosymmetry to asymmetric structures [17]. Symmetric structures consist of two identical excitation transfer pathways [18,19,20]. The most current photosynthetic organisms use pseudosymmetric reaction centers, and several experiments showed that one of the two excitation pathways is preferred over the other, regardless of their nearly identical structure [21].

**Figure 1:**
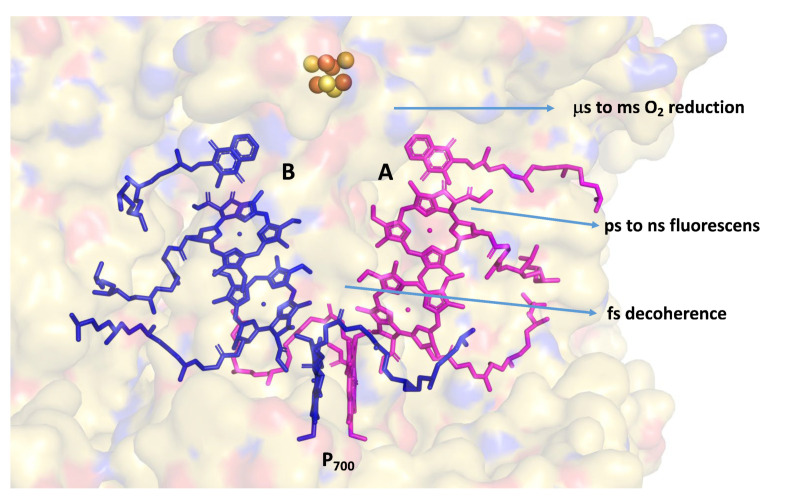
Two branches of electron transfer in PSI. Slips ranging from femtoseconds to microseconds are indicated. The structure of the prosthetic groups was taken from PDB 518R. Branch A (magenta), located in PsaA, and Branch B (blue), located in PsaB, which are in surface presentation at 80% transparency. It is apparent that Branch A is in close proximity to the membrane bulk, as well as to the aqueous environment.

We suggest that the primary evolutionary impetus for the emergence of pseudosymmetric reaction centers was the colonization of ecological niches exposed to high light intensities, a condition that proved fatal for the original organisms. In this way, one of the evolved excitation branches became more involved in slips, protecting the integrity of the reaction center. Homodimeric reaction centers are prevalent in anaerobic organisms such as Chlorobium, which grow under extremely low light intensities. Under these conditions, the excitation frequency of their reaction centers is expressed in seconds or even minutes [22]. Therefore, the existence of quantum mechanical slips is not necessary for protecting the reaction centers from photochemical damage. Even in PSI, which operates with close to 100% quantum efficiency, the two excitation branches are not equal in sharing the activity [23]. This is contrary to Photosystem II reaction centers that operate under high light intensities; the excitation takes place in a sub-nanosecond time course, but the two-step QB reduction takes milliseconds [24]. Consequently, in Photosystem II (PSII) reaction centers, only one branch is active in light-induced charge separation, while the other branch is either idle or involved in annihilation processes [25,26]. Thus, early in evolution, photosynthetic reaction centers established mechanistic slips to protect themselves from radiation damage. These slips operate on timescales ranging from femtoseconds to milliseconds and are supported by several other energy-dependent protective mechanisms. We propose that the establishment of protective mechanisms is the primary driving force in the evolution of photosynthetic reaction centers. Figure 1 depicts some of the protective slips in the case of Photosystem I (PSI) from the green lineage. It has been reported that the preferred excitation pathway in PSI of plants and green algae is Branch B [27,28,29]. Therefore, we place the slip-related excitation pathway on Branch A. This suggestion is supported by the fact that the isoprenoid side chain of the quinone in Branch A changes its position from one organism to another and even within the same species [30,31]. The structure of PSI complexes clearly shows that Branch A is more exposed to the membrane environment than Branch B [32,33]. Moreover, the position of the phytol chains and amino acids around the Branch B quinone is much more conserved than in Branch A. This situation strongly suggests that Branch B evolved to provide maximum coupling, while Branch A evolved to support slippage.

**Figure 2:**
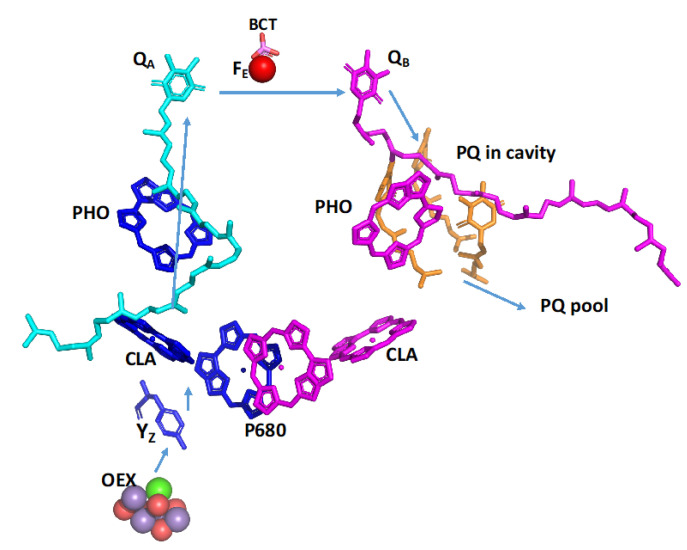
Cofactors involved in water oxidation and plastoquinone reduction in PSII. The electron transfer pathways are indicated by cyan arrows. Branch A is in blue, and Branch B is in magenta. Specific cofactors are indicated. Three plastoquinone molecules (brown) that may occupy the hydrophobic outlet cavity are indicated [34]. The coordinates were taken from PDB 8BD3.

While PSI operates via single electron events, PSII operates via two-electron reduction and four-electron oxidation steps. This constraint resulted in over 99% efficiency of light-induced charge separation in PSI and about 85% efficiency in PSII. The stability of oxidized plastocyanin and reduced ferredoxin extended the time course for handling PSI overoxidation or over-reduction from seconds to minutes. On the other hand, PSII must cope with potentially damaging events on the micro-to-millisecond timescale. In this case, extensive mechanistic slips, including internal cyclic electron transport, have evolved, some of which are species-specific [34]. The mechanistic couplings and interactions within PSII are exceptionally intricate, leading to a quantum efficiency of approximately 85% in plastoquinone reduction. Investigations of the PSII core (containing only four chlorophylls and two pheophytins) have unveiled enduring quantum beats occurring on the picosecond timescale [35]. Coherence between the excitons initiating the two distinct charge separation pathways persists for over 500 fs, while coherence between the exciton and charge transfer states—representing the reactant and the product of the charge separation reaction, respectively—remains present for at least 1 ps. The coherence existing between vibronic and charge transfer states might play a pivotal role in facilitating multichannel transitions into the metastable charge-separated state, achieving nearly 100% quantum efficiency. This effect is localized in the vicinity of the four chlorophyll molecules involved in the formation of P680 [26]. However, when the excitation reaches the pheophytin, the symmetry must break down, resulting in coherence only in Branch A and complete decoherence in Branch B. Consequently, in intact PSII, the excitation, charge separation, and oxidation reduction become unique [26]. Two distinct excitation pathways are operating: the productive pathway (A), which goes from P680 to Q_A_, and the nonproductive pathway (B), which goes from P680 to pheophytin A409 (Figure 2). Remarkably, the two pheophytin molecules are placed at a similar distance to Q_A_ (9.7 Å), as is pheophytin A408 to Q_B_ (9.3 Å), yet the latter is not active in productive photoreduction [34]. This phenomenon could be attributed to the persistence of coherence in Branch A, while decoherence predominates in Branch B. Q_B_-related slips have been recognized for some time [36]. They are manifested by thermoluminescence and delayed light emission that can last from milliseconds to seconds. All of them are affected by reduced plastoquinone, but the mechanistic involvement of Q_B_, cyt b559, carotenes, and Kok’s transition states is not clear. Despite 3.5 billion years of evolution, a remedy for the inefficiency of the system could not be found. The coupling between excitation and electron transport must slowdown from a femtosecond timescale to a millisecond timescale for oxygen evolution and plastoquinone reduction. This unsolved complexity has resulted in a very high turnover of the D1 protein [36,37]. The rapid destruction of the D1 protein leads to disassembly, followed by the synthesis and reassembly of PSII. The root cause of this rapid destruction is not satisfactorily explained, despite numerous manuscripts and reviews. We consider this effect as a long-term thermodynamical slip, and its global consequence is discussed below.

## 3. The Biochemical Domain of Microsecond-to-Second Coupling and Slips

The biochemical domain of microsecond-to-second coupling and slips involves the formation of oxidized and reduced substances resulting from photochemical reactions, which can last up to microseconds [6]. The highly oxidized components, mainly associated with PSII, need to be neutralized to prevent damage, such as amino acid oxidation and protein damage. Carotenoids play a crucial role in these processes by deactivating triplet chlorophyll (^3^Chl*) and singlet oxygen (^1^O₂*) through radical quenching and electro-cycling, as described earlier [38]. In addition, more elaborate protection mechanisms have evolved to handle highly reduced components in an oxidizing environment, which dominates the Earth’s surface, where most photosynthetic biomass production occurs [39,40]. The majority of ATP and NADPH generated by the photosynthetic electron transfer chain (ETC) is used to reduce CO_2_. However, while water is always available as the electron donor for this reaction, the electron acceptor (CO_2_) is often limited or absent. To cope with these constraints and the fact that electron acceptors are independent of light absorption and excitation, numerous mechanistic slips have been developed to protect photosynthetic membranes [41,42]. Recently, we proposed an additional element that contributes to the efficiency and protection of PSII [34]. Our research identified a hydrophobic cavity within PSII that appears capable of housing up to five plastoquinone molecules, some of which are positioned close enough to allow for electron tunneling from QB at the sub-microsecond timescale. This discovery challenges the conventional understanding of the PSII mechanism, particularly the idea that a reduced QB departs from its binding site before acquiring the second electron, a process that typically takes about one millisecond. This proposal suggests that at least some of the reduced plastoquinones are generated within the hydrophobic cavity. If proven correct, this finding could have profound implications, potentially extending to the regulation of global enthalpy in living systems.

There exists a network of multiple interacting elements with varying reaction times ranging from milliseconds to weeks, continuously maintaining photosynthetic organisms within a narrow safety range between efficient light harvesting and photoprotection [43,44]. This network includes various mechanisms, such as non-photochemical quenching (NPQ), photorespiration, antioxidant systems, nitrogen and sulfur assimilation, and lipid biosynthesis. NPQ is employed by plants and algae to protect themselves from the adverse effects of high light intensity [43]. It involves the quenching of singlet excited state chlorophylls through enhanced internal conversion to the ground state. NPQ serves as a major millisecond slip that protects photosynthesis in environments where the absorption of light energy exceeds the capacity for light use, such as in the case of CO_2_ limitation.

Within the chloroplasts, there are two systems involved in dealing with over-reduction or excess electrons [44]. The first is photorespiration, which employs the luminal PTOX system to oxidize reduced plastoquinone (PQ), and the second is stromal superoxide dismutase, which accepts excess electrons from PSI. These two slippages are vital for balancing the photosynthetic electron transport. Additionally, numerous protective systems have evolved to optimize photosynthetic productivity, including scavenging systems for reactive oxygen species (ROS), which involve soluble antioxidants such as glutathione and ascorbate [43,44].

## 4. Second-to-Minute Coupling and Slips

Imperfections are necessary for the adaptation and smoothness of operation in many biological processes. Certain biochemical processes function by coupling extremely precise reactions with others that enjoy a high degree of freedom and generate beneficial slippages [7]. The electron transport generates a proton-motive force (PMF) across thylakoid membranes. This coupling is prone to slippages because the membrane is partially proton permeable. Arguably, nature selected for PMF over chemical coupling because of its potential slip, which automatically balances the system, preventing the overproduction of a high-energy state [45]. The electrochemical gradient of protons (∆μH+), which is formed across the thylakoid membrane by the reaction centers and protein complexes in the electron transport chain, is prone to losses through leaks and slips. While leaks result from the nature of the membrane that is partially proton permeable, slips are often introduced into the catalytic protein by natural pressure. Several processes evolved on the system level to cope with imbalances in the driving source and the translation progressions. Cyclic electron transport (CEF) is a prime example of such dichotomy. Photosynthetic electron flow operates in two modes: linear and cyclic. In CEF, electrons are recycled around Photosystem I. As a result, a trans-thylakoid proton gradient (ΔpH) is generated, leading to the production of ATP without the concomitant production of NADPH. The evolution of this process was necessary to balance ATP and NADPH production for the demand of metabolic processes [45]. Arguably, this slippage-prone reaction was selected over chemical coupling because of its built-in slippages, which allow for flexibility in the conversion processes.

**Figure 3:**
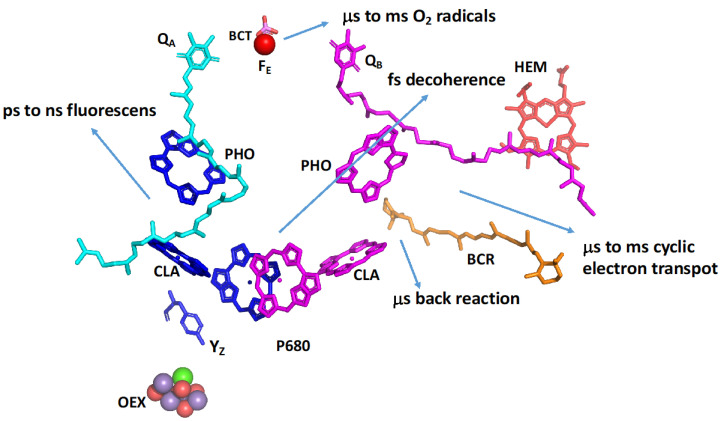
Multiple slippages that protect PSII from superfluous damages. Electron transfer Branch A is in blue. Branch B is in magenta. Branch B, together with the hem and carotene molecules, may function in cyclic electron transport and back-reaction that encompass several slip mechanisms. Decoherence results from absorbed photons being dissipated to heat. The coordinates were taken from PDB 8BD3.

State transition is another process that evolved at a high energetic expense for balancing processes. The state transition is a rapid physiological adaptation mechanism that adjusts the way absorbed light energy is distributed between Photosystem I and Photosystem II [46,47,48]. State transition involves rearrangements of the photosynthetic apparatus, which occur on short timescales of seconds to minutes. It was demonstrated that the exposure of several plant and green algae species to high light intensity results in the movement of LHCII complexes from PSII to PSI [48]. This protects the more damage-prone PSII and adds excitation energy to the resilient PSI that may be further protected by cyclic electron transport (Figure 3), as well as shuttling excess electrons to reductive processes and oxygen [49]. PSI is not totally resistant to excess excitation, and it may be further protected by modifying or eliminating subunits that are responsible for LHCII binding [50]. Recently, it was demonstrated that PSI is quite sensitive to fluctuating light intensities [51]. In addition, the formation of a stable PSI–PSII megacomplex in rice results in energy spillover [52]. Those three examples may be viewed as long-term physiological slips.

## 5. Minute-to-Eon Coupling and Slips

The long-lasting slips are governed by the second law of thermodynamics. The second law of thermodynamics is an expression of the universal law of increasing entropy, that is, a thermodynamic quantity representing the unavailability of a system’s thermal energy for conversion into mechanical work, often interpreted as the degree of disorder or randomness in the system [25,26,53]. Life is an expression of increased order; therefore, it requires constant energy input to be sustained. While the chemical energy of the Earth’s crust can supply limited global energy for sustaining life, the bulk of the energy comes from the Sun, and it is channeled to all living systems through photosynthesis.

During early evolution, around 3.5 billion years ago, the interplay between the Earth’s chemistry and photosynthesis was significant. At that time, Earth’s minerals and atmosphere maintained a low redox potential, and the oceans were rich in water-soluble reduced iron, one of the most abundant minerals on Earth. The onset of photosynthetic oxygen evolution brought about significant changes [54]. A large portion of the Sun’s energy, which is used to decrease entropy through the accumulation of organic matter, was redirected to oxidize iron, causing it to become insoluble and sediment. These orderly sediments have been stored underground and decreased global entropy. The accumulation of fossil fuels follows a similar principle, wherein their underground storage reduces global entropy on the Earth’s surface. Arguably, human activities, particularly the utilization of oxidized minerals and fossil fuels, have significantly disrupted the balance of entropy. These actions have led to an irreversible increase in disorder, driven by population growth and advancing lifestyles. Nearly all aspects of an advanced lifestyle are directly associated with global disorder. Nations universally aim to boost their Gross Domestic Product (GDP), a trend that contributes to the rise in global disorder. The unrestricted printing of money is directly linked to an increase in disorder since the surplus funds often go towards unnecessary leisure, further contributing to disorder. Low-cost flights, while popular, pose significant environmental challenges, and efforts to enhance their efficiency may not fully mitigate these issues. Additionally, the introduction of Bitcoin has generated billions of dollars, directly influencing the rise of global disorder.

Arguably, at a certain juncture in time, the global population and the standard of living reached a threshold beyond which the entropic equilibrium could no longer be sustained solely through photosynthesis. The identification of this specific time point is subjective. I propose that this critical juncture occurred in 1987, when the world population surpassed 5 billion, and lifestyle was considerably less extravagant compared with the present day. Consequently, it becomes imperative to revert back to the conditions of 11 July 1987 in terms of world population, energy consumption, automobile numbers, flight distances, and tangible productivity. Unfortunately, other than reductions in population and lifestyle changes, there are limited available methods to counteract the escalating global disorder. As a result, we are confronted with the consequences of an entropic trend that is progressively advancing toward greater disorder.

## 6. Harnessing Sunlight and Photosynthesis to Tip the Balance

Developing new technologies for generating clean and efficient energy is crucial for society to avert impending energy and environmental crises. Sunlight stands as the most abundant source of energy on the planet. Solar energy represents the most viable alternative to fully replace fossil fuels. It is imperative to shift from fossil fuel dependence towards a sustainable and clean energy economy capable of meeting the world’s growing energy demands. In recent years, there has been tremendous progress in photovoltaic production, especially in regions with ample sunlight, which may potentially cater to a significant portion of the energy needs. However, a primary challenge lies in the limitation of daily production time and the lack of effective technology for bulk energy storage to cover nighttime usage and cope with unfavorable climatic conditions. The current method of storage in rare metal-infested batteries is environmentally unsound. We proposed to use the Red Sea as an energy source for Europe and the Middle East [55]. It was suggested to damme the Bab al-Mandab and Suez Canal; consequently, the Red Sea level would drop by 8 m over a period of 4 years. The resulting gravitational potential energy could generate electricity while simultaneously being sustained by continuous evaporation. The construction of water steps along the dams could potentially support global maritime trade routes. This massive undertaking is estimated to generate around 1 TW of clean, sustainable, and consistent electricity. It is proposed to fulfill the entire nighttime energy demand of the Middle East and Europe. Nonetheless, it is crucial to acknowledge that such an initiative would present significant environmental, ecological, and geopolitical challenges. It would likely encounter considerable opposition due to its potential impact on the natural ecosystem.

Photosynthesis, utilizing sunlight, stands as the ultimate source of nearly all energy used in living organisms. The Sun supplies over 100,000 TW of light power to the Earth’s surface. Every pigment undergoes photochemical processes, including productive functions that generate new components and energy for various biological activities, including human processes. Photosynthesis has evolved to optimize these productive mechanisms. Presently, photosynthesis yields over 150 TW of chemical energy, encompassing both land and oceans. This amount surpasses the human global consumption of primary energy by tenfold. The idea of utilizing photosynthesis for energy production has been widely suggested, prompting numerous promises, often without a comprehensive account of the feasibility and consequences.

The solar energy stored through photosynthesis over billions of years constitutes the primary source of energy available on Earth. Among various technologies for hydrogen production, utilizing oxygenic natural photosynthesis holds substantial promise, as it would use clean and affordable sources of water and solar energy. However, achieving the global utilization of this process involves several key constraints that need consideration. The primary considerations include the spatial separation between oxygen evolution and hydrogen production. Operating with an energy efficiency close to the upper limit of oxygenic photosynthesis, approximately 5%, is crucial. Various methods have been proposed to achieve these objectives, yet none have met the desired targets thus far [55,56,57,58].

When devising a global solution to counteract the entropic deficit caused by burning fossil fuels, several constraints must be factored in: (1) The required installation area should cover several thousand square kilometers. (2) The sole utilization of seawater is permitted. (3) The selected organism must be robust and entirely resistant to bacterial and viral infections. (4) The energy value of the produced hydrogen must significantly surpass the energy (and monetary) investment required for constructing the installation. (5) Any excess organic material produced should be stored deep beneath the surface, aligning with the natural processes that led to the formation of fossil fuels over millions of years.

If all the aforementioned steps are implemented, they may aid in slowing down environmental damage; however, they are unlikely to entirely reverse the prevailing trend. From our perspective, it is crucial to devise an entirely new approach to mitigate the risk of global calamitous disorder. This approach needs to be as significant as iron sedimentation that reduced the global enthalpy over a billion years ago.
